# Magnesium Hydride Ameliorates Endotoxin-Induced Acute Respiratory Distress Syndrome by Inhibiting Inflammation, Oxidative Stress, and Cell Apoptosis

**DOI:** 10.1155/2022/5918954

**Published:** 2022-04-26

**Authors:** Xuan Shi, Lina Zhu, Sheng Wang, Wanli Zhu, Quanfu Li, Juan Wei, Di Feng, Meiyun Liu, Yuanli Chen, Xuejun Sun, Hongtao Lu, Xin Lv

**Affiliations:** ^1^Department of Anesthesiology, Shanghai Pulmonary Hospital, School of Medicine, Tongji University, Shanghai, China; ^2^Department of Naval Medicine, Naval Medical University, Shanghai, China; ^3^Center of Hydrogen Science, Shanghai Jiao Tong University, Shanghai, China

## Abstract

Acute respiratory distress syndrome (ARDS) causes uncontrolled pulmonary inflammation, resulting in high morbidity and mortality in severe cases. Given the antioxidative effect of molecular hydrogen, some recent studies suggest the potential use of molecular hydrogen as a biomedicine for the treatment of ARDS. In this study, we aimed to explore the protective effects of magnesium hydride (MgH_2_) on two types of ARDS models and its underlying mechanism in a lipopolysaccharide (LPS)-induced ARDS model of the A549 cell line. The results showed that LPS successfully induced oxidative stress, inflammatory reaction, apoptosis, and barrier breakdown in alveolar epithelial cells (AEC). MgH_2_ can exert an anti-inflammatory effect by down-regulating the expressions of inflammatory cytokines (IL-1*β*, IL-6, and TNF-*α*). In addition, MgH_2_ decreased oxidative stress by eliminating intracellular ROS, inhibited apoptosis by regulating the expressions of cytochrome c, Bax, and Bcl-2, and suppressed barrier breakdown by up-regulating the expression of ZO-1 and occludin. Mechanistically, the expressions of p-AKT, p-mTOR, p-P65, NLRP3, and cleaved-caspase-1 were decreased after MgH_2_ treatment, indicating that AKT/mTOR and NF-*κ*B/NLRP3/IL-1*β* pathways participated in the protective effects of MgH_2_. Furthermore, the in vivo study also demonstrated that MgH_2_-treated mice had a better survival rate and weaker pathological damage. All these findings demonstrated that MgH_2_ could exert an ARDS-protective effect by regulating the AKT/mTOR and NF-*κ*B/NLRP3/IL-1*β* pathways to suppress LPS-induced inflammatory reaction, oxidative stress injury, apoptosis, and barrier breakdown, which may provide a potential strategy for the prevention and treatment of ARDS.

## 1. Introduction

Acute respiratory distress syndrome (ARDS) is a harmful host response, which is caused by various conditions such as sepsis, severe trauma shock, pancreatitis, or inhalation of poisonous gases [1]. ARDS remains the leading cause of morbidity and mortality (about 40%) in septic patients [2, 3]. ARDS is characterized by disruption of the alveolar endothelial and epithelial barrier [4, 5], which in turn leads to pulmonary alveolar and interstitial edema, impaired gas exchange, and hypoxemia [6]. What's more, enhanced inflammation and oxidative stress are the pathologic hallmarks of ARDS [7]. The mechanism of ARDS remains unclear due to multiple cell types in the lung tissue, including endothelial cells, fibroblasts, and macrophages. At present, no effective pharmacotherapy is available to improve the survival rate of ARDS patients. In addition, ARDS survivors often suffer irreversible physical impairments, which seriously affect their quality of life [8]. Although many pharmacological therapies such as glucocorticoids, surfactants, protease inhibitors, and anti-inflammatory, antithrombotic, and fibrinolytic treatments have been attempted, none of them has proven to be fully effective [9–11]. Therefore, it is necessary to seek more effective treatments for ARDS by clarifying the underlying molecular mechanism.

Hydrogen gas (H_2_) is a novel antioxidant, which was first reported by Ohsawa et al. in 2007 as being able to alleviate oxidative stress by suppressing hydroxyl radicals (•OH) and peroxynitrite (ONOO-) [12]. Hydrogen has been reported to possess antioxidative, anti-inflammation, and antiapoptosis effects [13] and demonstrated as a novel therapy for different diseases such as cerebral, myocardial, hepatic, renal, and intestinal diseases [14–20]. Especially, hydrogen inhalation can alleviate hypertoxic lung injury in rats [21] and protect mice against cigarette-induced chronic obstructive pulmonary disease (COPD) [22]. Hydrogen has been reported to reduce the cytokine storm and oxidative stress reactions in mice [23, 24].

All the animal models in the above studies were treated with hydrogen mainly through three ways: hydrogen-rich water, hydrogen-rich normal saline (NS), and H_2_. As the hydrogen concentration in tissues depends on the administration route [25], the amount of H_2_ in hydrogen-rich water is limited. In this study, we used the magnesium hydride (MgH_2_), a promising hydrogen source with high hydrogen-storage capacity (7.6 wt%), to treat ARDS. MgH_2_ can produce a desired quantity of H_2_ following hydrolysis reaction at room temperature: MgH_2_ +2H_2_O → Mg(OH)_2_ +2H_2_. As the by-product is poison-free, it is possible to make use of MgH_2_ for biological application in clinical practice [26].

The aim of this study was to investigate the role of MgH_2_ in the pathophysiology of oxidative stress-mediated inflammation and apoptosis in ARDS, and provide evidence-based clues for use of MgH_2_ as a novel therapeutic target against ARDS.

## 2. Materials and Methods

### 2.1. Animals and Grouping

Male specific-pathogen-free (SPF) C57B/L6 mice weighing 20-25 g (Shanghai Lex Experimental Animal Center, Shanghai, China) were raised in SPF conditions. Animal care and experiments were approved by Shanghai Pulmonary Hospital (Shanghai, China) and performed in accordance with the Guide for the Care and Use of Laboratory Animals of the National Institutes of Health (NIH publications NO.8023, revised 1978). We used two types of ARDS models to detect the effects of MgH_2_ and Mg(OH)_2_ in alleviating ARDS. Firstly, mice were treated with intraperitoneal (i.p.) injection of LPS. Mice were used to observe survival rates in these four groups (CON group, treated with saline only; LPS group, treated with lipopolysaccharide, 15 mg/kg, i.p.; LPS+MgH_2_ group, treated with MgH_2_ at a daily dose of 50 mg/kg for 3 consecutive days before LPS injection; LPS+Mg(OH)_2_ group, treated with Mg(OH)_2_ at a daily dose of 110 mg/kg for 3 consecutive days before LPS injection to keep the concentration of Mg^2+^ ion consistent in different groups) and LPS (10 mg/kg, i.p.) was used for the other experiments. Secondly, mice were treated with LPS intratracheally (5 mg/kg, i.t.) using a MicroSprayer syringe assembly (MSA-250-M, Penn Century, USA) as previously described [27] under anesthesia with 0.75% intraperitoneal pentobarbital (75 *μ*g/g). MgH_2_ and Mg(OH)_2_ were treated as previously described.

### 2.2. Cell Culture and Drug Treatment

Alveolar epithelial cell line A549 (Shanghai Institutes for Biological Sciences of the Chinese Academy of Sciences, Shanghai, China) was grown in Dulbecco's Modified Eagle's Medium (DMEM) (Gibco, CA, USA) with 10% fetal bovine serum (FBS; Gibco, CA, USA) and 1% penicillin and streptomycin (Thermo Fisher Scientific, Waltham, MA, USA) at 37°C in 95% air and 5% CO_2_.

500 ng/ml LPS stimulation was used to establish the ARDS model. To determine the optimal MgH_2_ concentration, A549 cells were treated with 50 *μ*M, 100 *μ*M, 200 *μ*M, 500 *μ*M, 1000 *μ*M, and 2000 *μ*M MgH_2_ (Kemike, Wuhan, China). Finally, 500 *μ*M was chosen as the appropriate concentration for the subsequent experiments. 5 mM N-Acetyl-L-cysteine (NAC) was added into cells as a positive control.

### 2.3. Cell Viability Assay

A549 cells were seeded into a 96-well plate at 5 × 10^3^ cells/ml, with addition of 100 *μ*l culture medium to each well. After 24-hour pre-incubation in a cell incubator, 10 *μ*l various concentrations of MgH_2_ were added to the plate, with 3 wells for each concentration. 10 *μ*l CCK8 reagent (Dojindo, Japan) was subsequently added into each well for 2-hour incubation. The optical density (OD) value was detected at the wavelength of 450 nm in a microplate reader. The whole process was repeated for 3 times.

### 2.4. RNA Extraction and Real-Time Quantitative PCR (RT-qPCR)

Total RNA was extracted from A549 cells and the lung tissues using Trizol reagent (Invitrogen, CA, USA) according to the manufacturer's instruction. The RNA concentration and purity were determined by 260/280 nm absorbance. Complementary DNA (cDNA) was reversed by reverse transcription kits (Vazyme, China). SYBR Green PCR kits (Yeasen, China) were used for RT-qPCR. The primers are listed in Table S1, and the primers for RT-qPCR were synthesized by Shanghai Sangon Biotech Co., Ltd (Shanghai, China). The expression levels of all genes were normalized with the level of *β*-actin in the same group.

### 2.5. Western Blot

Western blot analysis was performed as previously described [28]. Cells were collected and washed twice with phosphate-buffered saline (1 × PBS), and then lysed in cytoplasmic protein extraction reagent A supplemented with 1 mM PMSF. Lung tissues were collected 24 hours after LPS injection. The protein concentrations of all samples were determined using a BCA protein assay kit (Thermo Fisher Scientific, San Jose, CA, USA). Total protein was separated by 10% SDS-PAGE gel and then transferred to a polyvinylidene fluoride (PVDF) membrane (BioRad, Hercules, California, USA). The membrane was blocked with 5% non-fat dry milk in Tween 20/Tris-buffered saline (TBST) for 2 hours at room temperature and incubated with primary antibodies overnight at 4°C. The antibodies used for Western blotting are as follows: *β*-actin (1 : 1000, Proteintech, Chicago, USA), Bax (1 : 1000, Cell Signaling Technology, Beverly, MA, USA), Bcl-2 (1 : 1000, Cell Signaling Technology, Beverly, MA, USA), cytochrome c (1 : 1000, Cell Signaling Technology, Beverly, MA, USA), occludin (1 : 1000, Cell Signaling Technology, Beverly, MA, USA), ZO-1 (1 : 1000, Cell Signaling Technology, Beverly, MA, USA), NLRP3 (1 : 1000, Cell Signaling Technology, Beverly, MA, USA), caspase-1 (1 : 1000, Cell Signaling Technology, Beverly, MA, USA), cleaved-caspase-1 (1 : 1000, Cell Signaling Technology, Beverly, MA, USA), cleaved-caspase-3 (1 : 1000, Cell Signaling Technology, Beverly, MA, USA), IL-1*β* (1 : 1000, Cell Signaling Technology, Beverly, MA, USA), AKT (1 : 1000, Cell Signaling Technology, Beverly, MA, USA), p-AKT (1 : 1000, Cell Signaling Technology, Beverly, MA, USA), mTOR (1 : 1000, Cell Signaling Technology, Beverly, MA, USA), p-mTOR (1 : 1000, Cell Signaling Technology, Beverly, MA, USA), P65 (1 : 1000, Cell Signaling Technology, Beverly, MA, USA), p-P65 (1 : 1000, Cell Signaling Technology, Beverly, MA, USA). The membrane was washed with TBST three times, incubated with secondary antibodies (Licor, USA) for 2 hours at room temperature, washed again three times with TBST, and finally detected for fluorescence signal using the Odyssey Fluorescence Imaging System (Gene, USA). The expression level of *β*-actin was used as an internal control.

### 2.6. Inflammatory Cytokine Assay

For in vitro study, the supernatant was collected for inflammatory cytokines assay. For in vivo study, bronchoalveolar lavage fluid (BALF) was collected from the mice of all groups to measure the levels of IL-1*β*, IL-6, and TNF-*α* using the ELISA kits (Abcam, USA) according to the manufacturer's instructions.

### 2.7. Measurement of Intracellular Reactive Oxygen Species (ROS), Mitochondrial Membrane Potential (m∆*ψ*), and mitoSOX

Intracellular ROS was detected with the ROS assay kit (Beyotime, China) according to the manufacturer's protocol using the fluorescence imaging system (Bio-Real, Austria). m∆*ψ* was visualized with the JC-1 staining assay kit according to the manufacturer's protocol (Beyotime, China). Changes of m∆*ψ* were expressed by fluorescence intensity and analyzed by the ratio of aggregated JC-1 and monomeric JC-1. MitoSOX was detected with the MitoSOX assay kit (Yeasen, China) according to the manufacturer's protocol.

### 2.8. Histology and Immunohistochemistry

A portion of the lung tissue was fixed in 4% paraformaldehyde for 24 hours, paraffin embedded, sliced into 5 *μ*m sections, and stained with hematoxylin and eosin (H&E). For immunohistochemistry of NLRP3, IL-1*β*, and 8-Oxoguanine DNA Glycosylase (8-oxo-dG), sections were incubated at 4°C overnight with primary antibodies. The antibodies used are as follows: NLRP3 (1 : 100, Cell Signaling Technology, Beverly, MA, USA), IL-1*β* (1 : 100, Cell Signaling Technology, Beverly, MA, USA), 8-oxo-dG (1 : 100, Thermo Fisher Scientific, San Jose, CA, USA). The sections were washed with PBS, incubated in HRP-tagged goat anti-rabbit antibody (1 : 500, Proteintech, Chicago, USA) at 37°C for 1 hour. The nuclei were stained with DAPI reagents.

### 2.9. Lung Injury Analysis and Lung Tissue Wet-to-Dry Weight (W/D) Ratio Analysis

The severity of lung damage was scored based on the following histologic features, as has been described previously [27]. The W/D ratio was calculated by measuring the wet weights of the lung tissues after lung injury and the dry weights were measured 24 hours after placing the lung tissue in an 80°C oven at three time points until the weights remained unchanged, based on which pulmonary edema was evaluated.

### 2.10. Reactive Oxidative Stress Activity Assay

The levels of malondialdehyde (MDA), glutathione (GSH), and the activity of superoxide dismutase (SOD) in the mouse lung tissues were measured with the assay kits (Jiancheng Institute of Biotechnology, Nanjing, China) following the manufacturers' protocols.

### 2.11. TUNEL Staining of Lung Tissue

Apoptosis cells in the paraffin-embedded sections were stained by terminal deoxynucleotidyl transferase-mediated dUTP nick-end labeling (TUNEL) using a kit as previously described [29], followed by counterstaining with 4′6-diamidino-2-phenylindole (DAPI) for the nuclei. The experiment was performed according to the manufacturer's instructions (BioVision). The staining was observed under a fluorescence microscope (Olympus Corporation, magnification). TUNEL-positive cells were defined as cells with green staining (wavelength, 488 nm).

### 2.12. Statistical Analysis

All experiments were performed with independent three replicates. The experimental data were presented as the mean ± standard deviation (SD). Differences were analyzed by SPSS 17.0 statistical software with one-way ANOVA followed by Tukey's post hoc test. Differences were determined to be statistical significance if *P* < 0.05. Corresponding significances were indicated in the figures.

## 3. Results

### 3.1. MgH_2_ Attenuates LPS-Induced ARDS and Improves the Survival Rate

**Figure 1:**
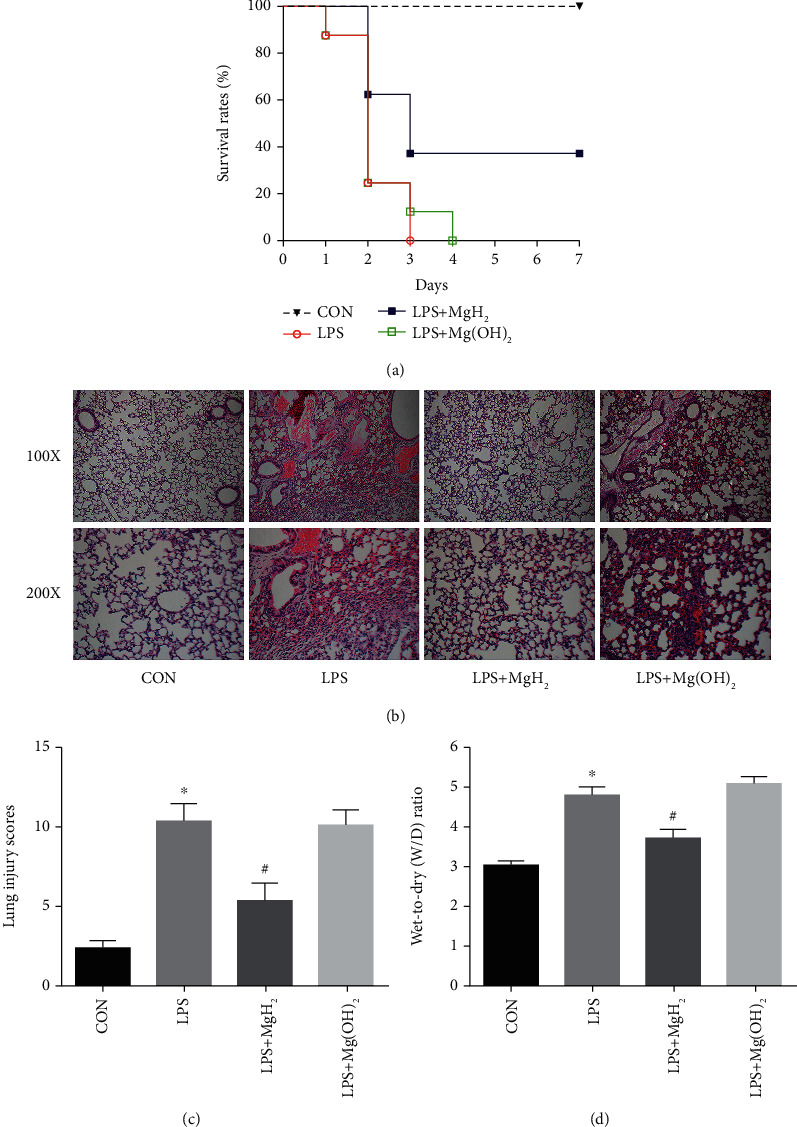
MgH_2_ attenuates ARDS and improves the survival rate of endotoxemia mice. (a). Survival curves of mice in different groups. (b). The H＆E staining of lung tissues (100x and 200x). (c). The lung injury score of mice in different groups. (d). The lung W/D ratio. Data are presented as mean ± SD. ^∗^*P* < 0.05 vs. the CON group, ^#^*P* < 0.05 vs. the LPS group.

LPS delivered at a dose of 15 mg/kg, killed 100% of mice within 3 days. Compared with LPS group, the survival rate of endotoxic mice treated with MgH_2_ (50 mg/kg) was significantly improved to almost 40% (*P* < 0.05) (Figure 1(a)). We further observed the protective effect of MgH_2_ on LPS-induced ARDS using H&E staining, and found that lung injury was alleviated after MgH_2_ administration as compared with LPS group (Figure 1(b)). Severe lung hemorrhage, lung edema, inflammatory cell infiltration, and thickening of the alveolar septa were observed in LPS group, all of which were alleviated after MgH_2_ administration. Consistently, the lung injury score was largely reduced in mice pretreated with MgH_2_ (Figure 1(c)), and the wet-to-dry (W/D) ratio of the lung tissues was decreased in LPS+MgH_2_ group (Figure 1(d)).

Then, we used another ARDS model (LPS, 5 mg/kg, i.t.) to detect the protective effect of MgH_2._ H&E staining, the lung injury score, and the W/D ratio of the lung tissues were also analyzed. As shown in Figure S1, after LPS intratracheal injection, the images of the lung showed severe lung hemorrhage, lung edema, inflammatory cell infiltration, and thickening of the alveolar septa. The lung injury score and W/D ratio were increased by LPS. However, the administration of MgH_2_ attenuated lung injury.

### 3.2. Effects of MgH_2_ on Oxidative Stress in AEC Damage following LPS Exposure

**Figure 2:**
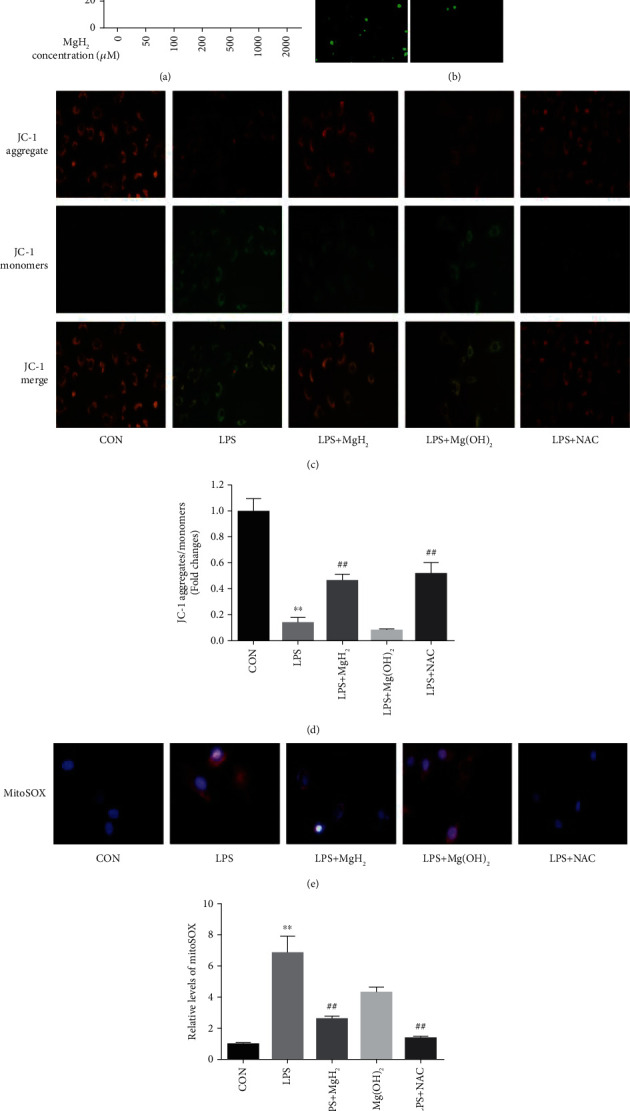
Effects of MgH_2_ on oxidative stress in (AEC) damage following LPS exposure. (a) The cell viability of A549 cells after MgH_2_ treatment. Data are presented as mean ± SD. ^∗^*P* < 0.05 vs. 0 *μ*M MgH_2_ group, ^∗∗^*P* < 0.01 vs. 0 *μ*M MgH_2_ group. (b) Effects of MgH_2_ on the levels of intracellular ROS (400x) by fluorescence microscope. (c) and (d) Effects of MgH_2_ on the cellular mitochondrial membrane potential in A549 cells detected with JC-1 solution (400x). (e) and (f) Effects of MgH_2_ on the levels of mitochondrial superoxide (mitoSOX) in A549 cells with LPS stimulation (400x). Data are presented as mean ± SD. ^∗∗^*P* < 0.01 vs. the CON group, ^##^*P* < 0.01 vs. the LPS group.

No significant changes in cell proliferation were observed when the concentration of MgH_2_ was lower than 500 *μ*M (Figure 2(a)), so we chose 500 *μ*M as the MgH_2_ concentration for the cell experiments. Oxidative stress response is one of the causes for the high mortality of ARDS. To understand the underlying mechanism of MgH_2_ in alleviating oxidative stress response and relieving ARDS, we used MgH_2_ to treat LPS-induced AEC damage by detecting the amount of intracellular ROS and used NAC as a positive control, which is known as an effective ROS remover. The results showed that the generation of ROS in the LPS-induced AEC damage model was suppressed after MgH_2_ treatment via DCFH-DA staining with immunofluorescence detection, in NAC group, the expression of ROS was also decreased (Figure 2(b)). Next, we used the mitochondrial function as the marker of oxidative stress [30] and found that MgH_2_ also alleviated the ratio of JC-1 aggregates/monomers (Figures 2(c) and 2(d)) and the expression of mitoSOX (Figures 2(e) and 2(f)) in the damage model caused by LPS in AEC as what were detected in NAC group.

### 3.3. Effects of MgH_2_ on Inflammatory Response and Cell Apoptosis in AEC Damage following LPS Exposure

**Figure 3:**
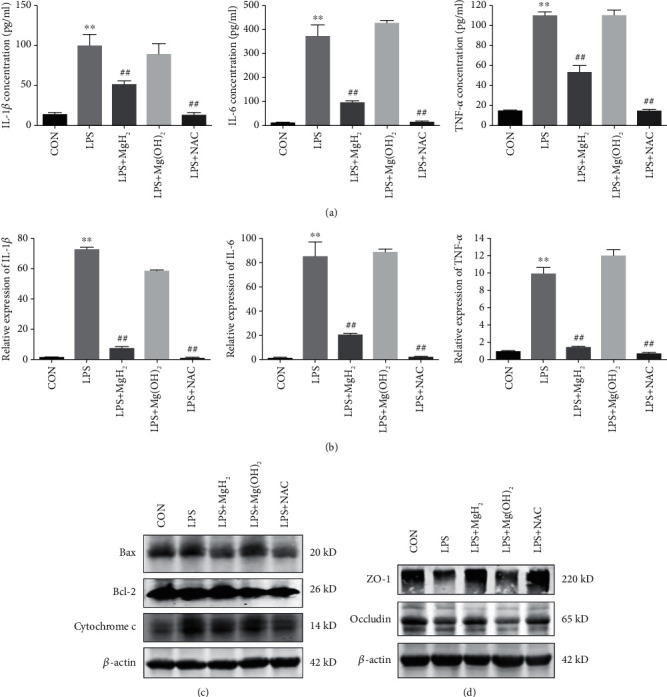
Effects of MgH_2_ on inflammatory response and cell apoptosis in AEC damage following LPS exposure. (a) and (b). The effects of MgH_2_ on inflammatory cytokine levels in LPS-stimulated A549 cells. (c) The effects of MgH_2_ on critical apoptosis-related proteins Bax, Bcl-2, and cytochrome c expressions in LPS-stimulated A549 cells. (d) MgH_2_ alleviated damage to the barrier-related proteins occludin and ZO-1 in LPS-stimulated A549 cells. Data are presented as mean ± SD. ^∗∗^*P* < 0.01 vs. CON group, ^##^*P* < 0.01 vs. LPS group.

Inflammatory cytokines (IL-1*β*, IL-6, and TNF-*α*) were detected as inflammatory response markers for the anti-inflammatory effects of MgH_2_ in AEC (Figures 3(a) and 3(b)). NAC was used as a positive control and the effects of MgH_2_ on the inflammatory response were similar to those of NAC. In addition, critical apoptosis-related proteins Bax, Bcl-2, and cytochrome c were detected to show the anti-apoptotic effect of MgH_2_ (Figure 3(c)). Occludin and ZO-1 are known as the major components of the tight junction in the AEC surface to prevent the leakage of tissue fluid into the alveolar space [31]. It was found that MgH_2_ treatment also alleviated damage to the barrier (Figure 3(d)) in LPS-induced AEC damage.

### 3.4. Effects of MgH_2_ on LPS-Induced Oxidative Stress in Lung Tissues of Endotoxemia Mice

**Figure 4:**
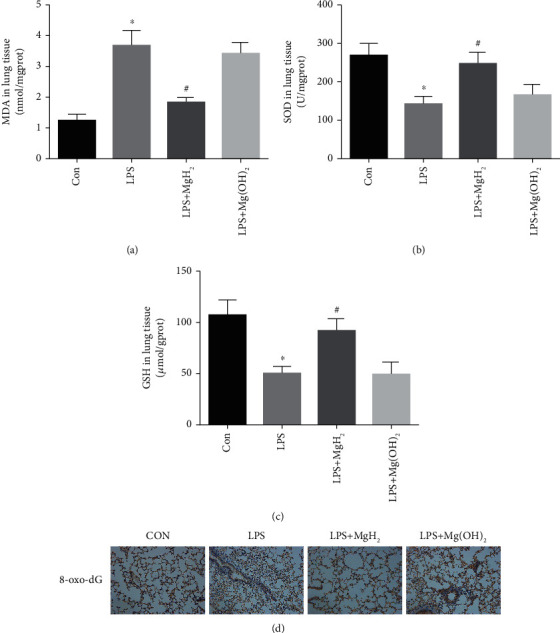
Effects of MgH_2_ on LPS-induced oxidative stress in lung tissues of endotoxemia mice. (a)–(c) The levels of MDA, SOD, and GSH in mice lung tissues in different groups. (d) 8-oxo-dG immunohistochemistry staining in mice lung tissues in different groups (200X). Data are presented as mean ± SD. ^∗^*P* < 0.05 vs. the CON group, ^#^*P* < 0.05 vs. the LPS group.

To clarify the protective effect of MgH_2_ on ARDS, MDA, SOD, and GSH levels were detected in the lung tissues. It was found that MDA in LPS group was significantly elevated and the levels of SOD and GSH showed the opposite trend. MgH_2_ treatment reversed these oxidative stress markers (Figures 4(a)–4(c)). We next detected 8-oxo-dG, the enzyme responsible for the excision of 8-oxoguanine. As expected, LPS increased 8-oxo-dG markedly, and this increasing trend was reversed after MgH_2_ treatment in vivo (Figure 4(d), Figure S2A).

### 3.5. Effects of MgH_2_ on Inflammatory Response and Cell Apoptosis in Lung Tissues in Endotoxemia Mice

**Figure 5:**
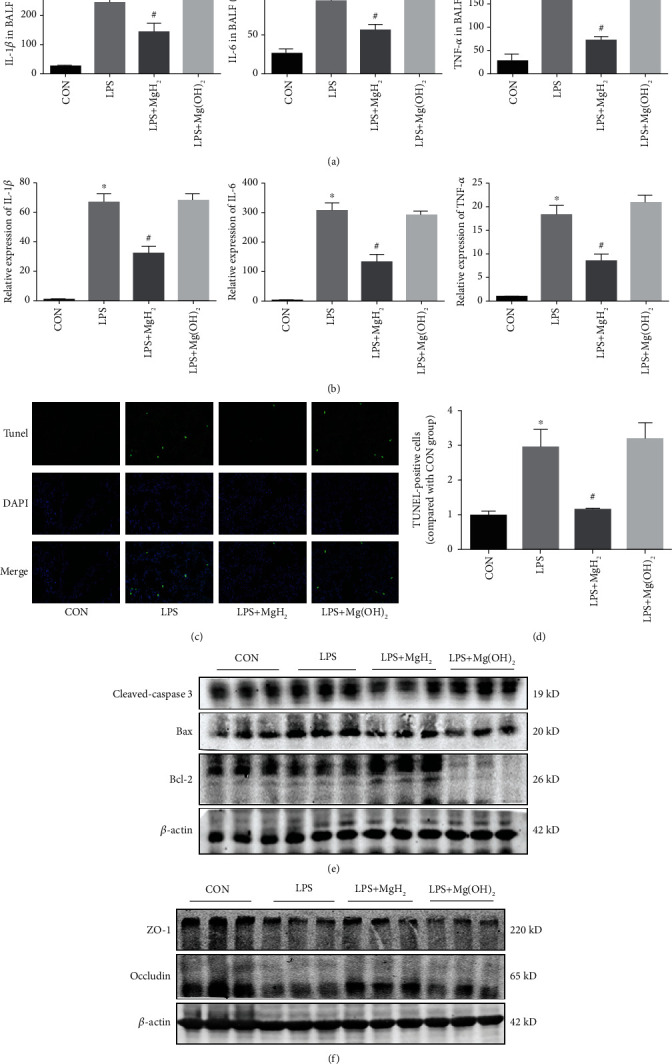
Effects of MgH_2_ on inflammatory response and cell apoptosis in lung tissues of endotoxemia mice. (a) Protein concentrations of IL-1*β*, IL-6, and TNF-*α* in BALF. (b) RT-qPCR analysis of IL-1*β*, IL-6, and TNF-*α* in the lung tissues. (c) and (d) The effects of MgH_2_ on cell apoptosis were tested by TUNEL staining in the lung tissues (200x). (e) and (f) The levels of critical apoptosis-related proteins and barrier-related proteins in the lung tissues. Data are presented as mean ± SD. ^∗^*P* < 0.05 vs. the CON group, ^#^*P* < 0.05 vs. the LPS group.

BALF were extracted from mice in all groups, and the levels of inflammatory cytokines in BALF were detected by ELISA in all groups. The concentrations of IL-1*β*, IL-6, and TNF-*α* in BALF (Figure 5(a)) and RT-qPCR analysis of IL-1*β*, IL-6, and TNF-*α* in lung tissues (Figure 5(b)) in LPS group were significantly higher than those in CON group, and MgH_2_ administration effectively suppressed these inflammatory cytokines.

Lung injury is known to be associated with cell apoptosis and barrier damage. TUNEL staining was used to determine the apoptosis of cells. It was found that a great number of positive staining points were distributed in LPS group. After MgH_2_ treatment, LPS-induced cell apoptosis was reduced as compared with LPS group (Figures 5(c) and 5(d)). Apoptosis-related proteins showed the same trends in two types of ARDS models (Figure 5(e), Figure S2B). Additionally, occludin and ZO-1 expressions were down-regulated after LPS stimulation, and these trends were reversed after MgH_2_ treatment (Figure 5(f), Figure S2C).

### 3.6. MgH_2_ Suppresses AKT/mTOR Pathway and NF-*κ*B/NLRP3/IL-1*β* Pathway in Vitro and in Vivo in LPS-Induced ARDS Models

**Figure 6:**
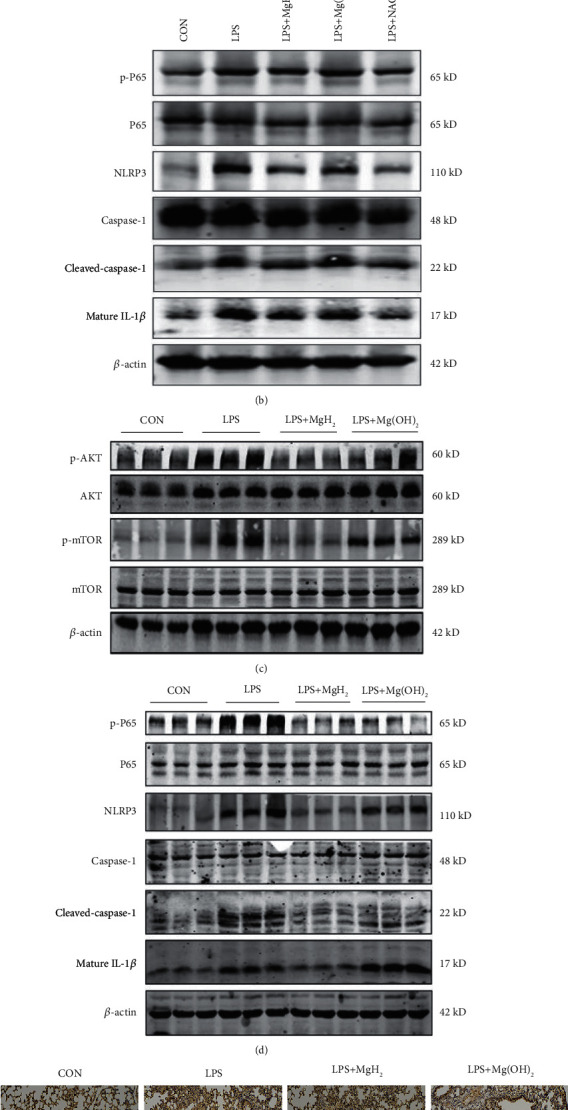
MgH_2_ suppresses AKT/mTOR pathway and NF-*κ*B/NLRP3/IL-1*β* pathway in vitro and in vivo. (a) and (b) MgH_2_ inhibits AKT/mTOR pathway and NF-*κ*B/NLRP3/IL-1*β* pathway related proteins in vitro; NAC is used as a positive control. (c) and (d) MgH_2_ inhibits AKT/mTOR pathway and NF-*κ*B/NLRP3/IL-1*β* pathway related proteins in vivo. (e) NLRP3 and IL-1*β* immunohistochemistry staining in lung tissues (100x).

The AKT/mTOR signaling pathway is activated in LPS-induced ARDS models in vitro and in vivo. NF-*κ*B transcription factors regulate the expressions of hundreds of genes involved in regulating oxidative stress, cell apoptosis, and inflammation [32]. In addition, NF-*κ*B is important for inflammasome priming and assembly [33] and the activation of NLRP3 plays a crucial role in the occurrence and development of ARDS [34]. To determine whether MgH_2_ suppressed oxidative stress, inflammation, cell apoptosis, and barrier breakdown via AKT/mTOR pathway and NF-*κ*B/NLRP3/IL-1*β* pathway, we used MgH_2_ to treat LPS-induced AEC and found that p-AKT, p-mTOR, and p-P65 were up-regulated in LPS group. The activation of inflammasome was accompanied with the up-regulation of NLRP3, cleaved-caspase-1, and mature IL-1*β* in LPS group. Of interest, MgH_2_ could significantly reduce the expressions of these proteins just as the effects of NAC (Figures 6(a) and 6(b)). The same experiments were conducted in vivo. It was found that the expressions of p-AKT and p-mTOR were increased in LPS group, and decreased after oral MgH_2_ administration. The similar trends were shown in the expressions of NLRP3, cleaved-caspase-1, and mature IL-1*β* (Figures 6(c) and 6(d), Figure S3A, B). What's more, the results of immunohistochemistry showed that the expressions of NLRP3 and IL-1*β* were obviously elevated in the lung tissues in LPS group, and reduced in LPS+MgH_2_ group (Figure 6(e), Figure S3C).

## 4. Discussion

ARDS is a common disease infected with Gram-negative bacteria containing LPS that affects millions of people worldwide. Inflammation, oxidative stress, and epithelial barrier impairment are the main characteristics of ARDS, leading to high mortality of ARDS patients [35]. Therefore, drugs with the effects of reducing inflammation, and oxidative stress and repairing the epithelial barrier function should be able to exert an unexpected therapeutic effect on ARDS. To the best of our knowledge, our study is the first one to investigate the effects of MgH_2_ in murine and cell models of ARDS.

In this study, we firstly showed these following findings: (1) MgH_2_ administration improved the survival rate of ARDS mice induced by LPS. (2) MgH_2_ administration reduced lung injury scores, alleviated lung edema, inflammatory cell infiltration, thickening of the alveolar septa, and inflammatory cytokines in ARDS mice. (3) MgH_2_ administration decreased oxidative stress and cell apoptosis and restored tight junctions in vitro and in vivo. (4) MgH_2_ regulated AKT/mTOR pathway and NF-*κ*B/NLRP3/IL-1*β* pathway in vitro and in vivo.

This study discovered that MgH_2_ administration improved the survival rate of LPS-induced ARDS mice and reduced inflammation in lung tissues. As described in the introduction, MgH_2_ can produce Mg(OH)_2_ and H_2_ following hydrolysis reaction in the stomach: MgH_2_ +2H_2_O → Mg(OH)_2_ +2H_2_. Mice treated with Mg(OH)_2_ showed no effects when compared with MgH_2_, which means H_2_ is the main cause of these changes in this study. Inflammation is a common process in many diseases, which promotes the excessive activation of the immune system and the release of several cytokines. A lot of research reported that molecular hydrogen therapy reduced the levels of inflammatory cytokines and increased anti-inflammatory cytokines [36–38]. These effects can be induced by reducing the number of inflammatory cells [14] and inhibiting the activity of ONOO- [39].

The study also found that MgH_2_ administration decreased oxidative stress in vitro and in vivo. Oxidative stress, the imbalance of oxidants and antioxidants, is the critical role in the process of ARDS. The excessive expression of oxidants such as ROS, which is generated by the electron leakage of the mitochondrial respiratory chain, causes the oxidative burst. Molecular hydrogen has been reported to alleviate oxidative stress in some ways. For one hand, hydrogen can selectively suppress •OH and ONOO- to alleviate oxidative stress [12]. For another hand, hydrogen can indirectly act on the antioxidant system to alleviate oxidative stress via enhancing antioxidant enzyme activity and antioxidant gene expression, repairing mitochondrial function [40, 41]. In this regard, our results were consistent with those of Naomi Kamimura et al. [37], who pointed out in their research that MgH_2_ reduced the expression of 4-HNE, which is regarded as a second messenger in oxidative stress signaling [42].

Furthermore, it was also revealed in our research that after MgH_2_ administration, the expressions of Bax and cleaved-caspase-3 were remarkably down-regulated while Bcl-2, ZO-1, and occludin were up-regulated notably. Apoptosis is found to be triggered off by two pathways, the intrinsic pathway is related to modulators within the cell itself and the extrinsic pathway is related to extracellular stimuli and apoptosis receptors on the cell membrane [43], the former of which is associated with cytochrome c, cleaved-caspase-3, Bax, and Bcl-2. Hydrogen was reported to protect DNA and proteins from free radicals and keep the normal mitochondrial functions to down-regulate apoptosis [44, 45]. In addition, occludin and ZO-1 are the components of the tight junctions to prevent the alveolar space from the leakage of tissue fluid [31]. As for tight junction, Yu et al. have shown that hydrogen can protect the blood-brain barrier by decreasing its permeability via increase ZO-1 and VE-cadherin expressions [46]. Hydrogen-rich saline was also reported to maintain the integrity of intestinal epithelial tight junction barrier in rats with intestinal ischemia-reperfusion injury [47] and protect lung microvasculature of mice from sepsis-induced endothelial dysfunction and maintain the coherence of pulmonary endothelium [48].

In the global breakout of COVID-19, ARDS is one of the characteristics of COVID-19 and lacks effective therapies [49]. In China, hydrogen-oxygen inhalation for the treatment of COVID-19 has been proposed in the “Diagnosis and Treatment Protocol for Novel Coronavirus Pneumonia (Trial Version 7&8)”. Guan et al. [50] showed the efficacy of hydrogen/oxygen mixed gas inhalation in patients with COVID-19 in a multicenter, open-label clinical trial. Yang et al. [51] and Wang et al. [52] reviewed hydrogen therapy in treatment against COVID-19. Results in our study may provide a potential approach to combat novel coronavirus pneumonia clinically.

Taking what has been stated above into consideration, we can draw the conclusion that MgH_2_ administration ameliorates endotoxin-induced acute respiratory distress syndrome by inhibiting inflammation, oxidative stress, and cell apoptosis, thus promising a new target for managing endotoxin-induced ARDS. Hydrogen has efforts against many diseases. However, there are still no effective ways to detect the diffusion of hydrogen in vitro and in vivo. Firstly, hydrogen diffuses rapidly and widely. Secondly, there are still no tracer methods to examine hydrogen distribution in the tissues and circulation. What's more, the direct targets of hydrogen remain unclear due to the rapid and extensive diffusion of hydrogen. More experimental and clinical research is required to detect the specific mechanism of hydric action in cells and mice. Therefore, more studies should be carried out and have the findings applied to benefit people with endotoxin-induced ARDS.

## 5. Conclusions

This study demonstrated that MgH_2_ alleviates ARDS in vitro and in vivo through regulating AKT/mTOR pathway and NF-*κ*B/NLRP3/IL-1*β* pathway. Besides, MgH_2_ plays an important role in alleviating LPS-induced ARDS by relieving lung edema and pulmonary histological damage, and suppressing the inflammatory response, oxidative stress, cell apoptosis, and barrier breakdown. All these findings suggest that MgH_2_ could be an effective and promising therapeutic option for the prevention of ARDS.

## Data Availability

We declare that the materials described in the manuscript, including all relevant raw data, will be freely available to any scientist who wishes to use them for noncommercial purposes, without breaching participant confidentiality.

## Supplementary Materials

Supplementary Materials Figure S1. MgH_2_ attenuates LPS-induced ARDS. A. Mice were treated with LPS intratracheally (LPS, 5 mg/kg). The H&E staining of lung tissues (100x and 200x). B. The lung injury score of mice in different groups. C. The lung W/D ratio. ^∗^*P* < 0.05 vs. the CON group, ^#^*P* < 0.05 vs. the LPS group. Figure S2. MgH_2_ attenuates LPS-induced oxidative stress and cell apoptosis in lung tissues of endotoxemia mice. A. 8-oxo-dG immunohistochemistry staining in the lung tissues (200x). B. The levels of critical apoptosis-related proteins in the lung tissues. C. The levels of critical barrier-related proteins in the lung tissues. Figure S3. MgH_2_ suppresses AKT/mTOR pathway and NF-*κ*B/NLRP3/IL-1*β* pathway in endotoxemia mice. A and B. MgH_2_ inhibits AKT/mTOR pathway and NF-*κ*B/NLRP3/IL-1*β* pathway related proteins in vivo. C. NLRP3 and IL-1*β* immunohistochemistry staining in the lung tissues (100x). Table S1. Primers used for reverse transcription-quantitative PCR.
