# Supplementary material for: Magnesium Hydride Ameliorates Endotoxin-Induced Acute Respiratory Distress Syndrome by Inhibiting Inflammation, Oxidative Stress, and Cell Apoptosis
Source: Oxid Med Cell Longev. 2022 Apr 26;2022:5918954. doi: 10.1155/2022/5918954 (PMC9072031; doi:10.1155/2022/5918954)
Supplement: Supplementary Materials — Figure S1. MgH2 attenuates LPS-induced ARDS. A. Mice were treated with LPS intratracheally (LPS, 5 mg/kg). The H&E staining of lung tissues (100x and 200x). B. The lung injury score of mice in different groups. C. The lung W/D ratio. ∗P < 0.05 vs. the CON group, #P < 0.05 vs. the LPS group. Figure S2. MgH2 attenuates LPS-induced oxidative stress and cell apoptosis in lung tissues of endotoxemia mice. A. 8-oxo-dG immunohistochemistry staining in the lung tissues (200x). B. The levels of critical apoptosis-related proteins in the lung tissues. C. The levels of critical barrier-related proteins in the lung tissues. Figure S3. MgH2 suppresses AKT/mTOR pathway and NF-κB/NLRP3/IL-1β pathway in endotoxemia mice. A and B. MgH2 inhibits AKT/mTOR pathway and NF-κB/NLRP3/IL-1β pathway related proteins in vivo. C. NLRP3 and IL-1β immunohistochemistry staining in the lung tissues (100x). Table S1. Primers used for reverse transcription-quantitative PCR. [file 5918954.f1.docx]

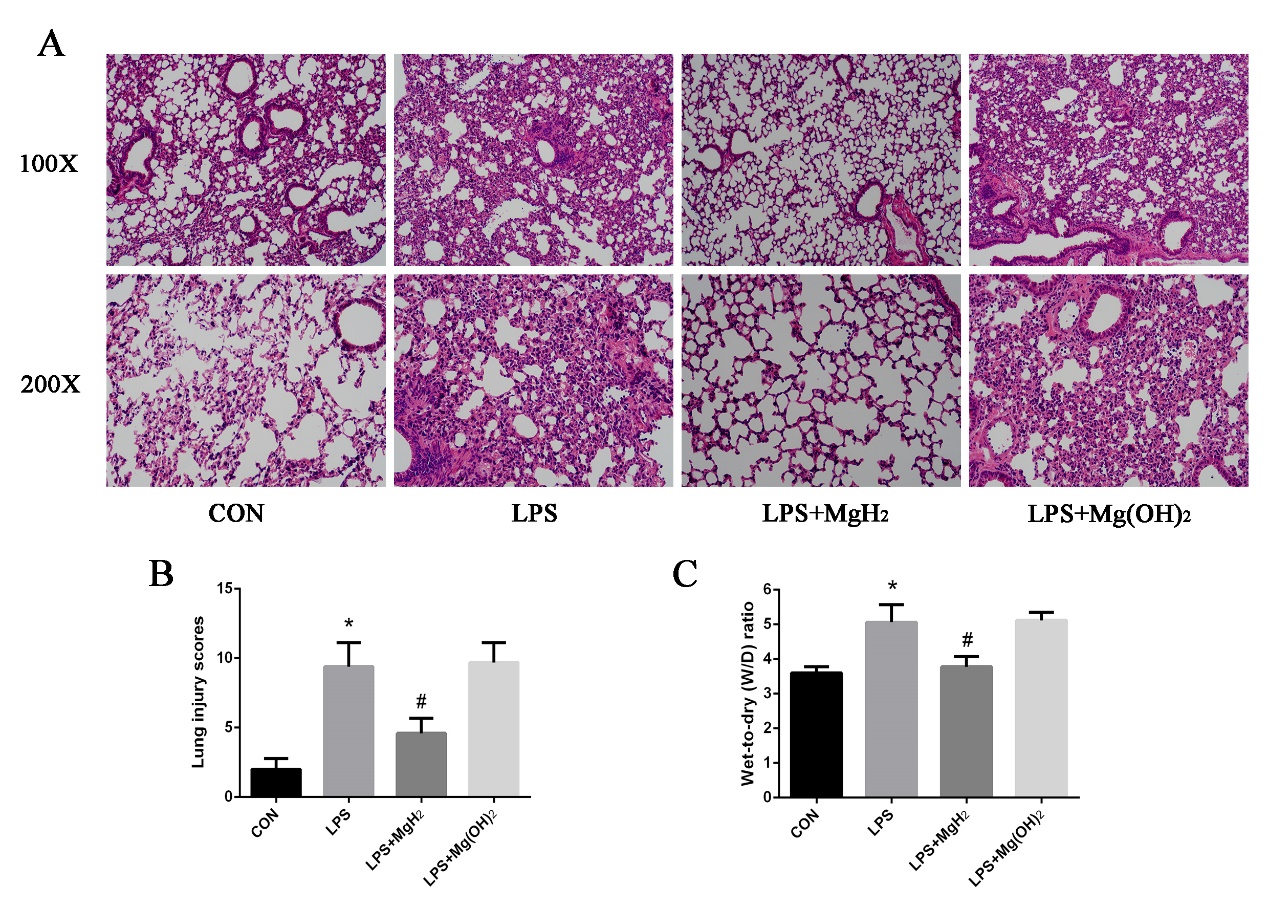


**Figure S1. MgH_2_ attenuates LPS-induced acute respiratory distress syndrome.** **A.** Mice were treated with LPS intratracheally (LPS, 5mg/kg). The haematoxylin and eosin (H&E) staining of lung tissues (100X and 200X). **B.** The lung injury score of mice in different groups. **C.** The lung W/D ratio. Data are presented as mean±SD. ^*^P<0.05 vs. the CON group, ^#^P<0.05 vs. the LPS group.


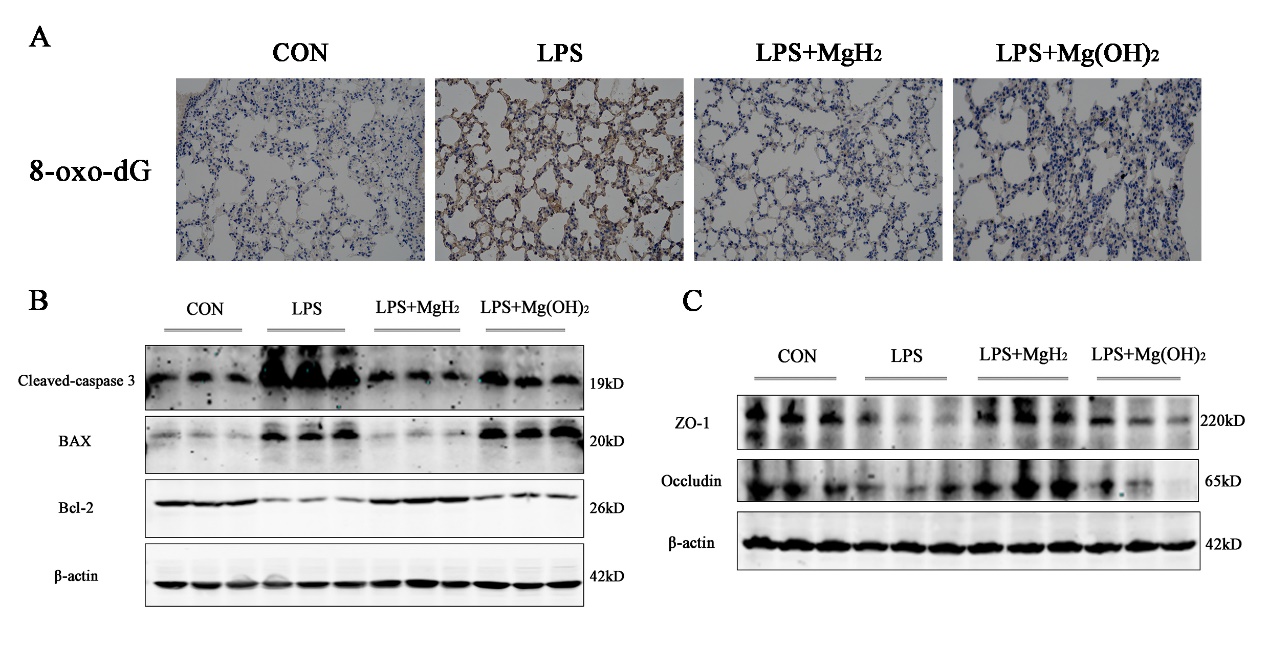


**Figure S2. MgH_2_ attenuates LPS-induced oxidative stress and cell apoptosis in lung tissues of endotoxemia mice. A.** 8-oxo-dG immunohistochemistry staining in mice lung tissues (200X). **B.** The levels of critical apoptosis-related proteins in the lung tissues. **C.** The levels of critical barrier-related proteins in the lung tissues.


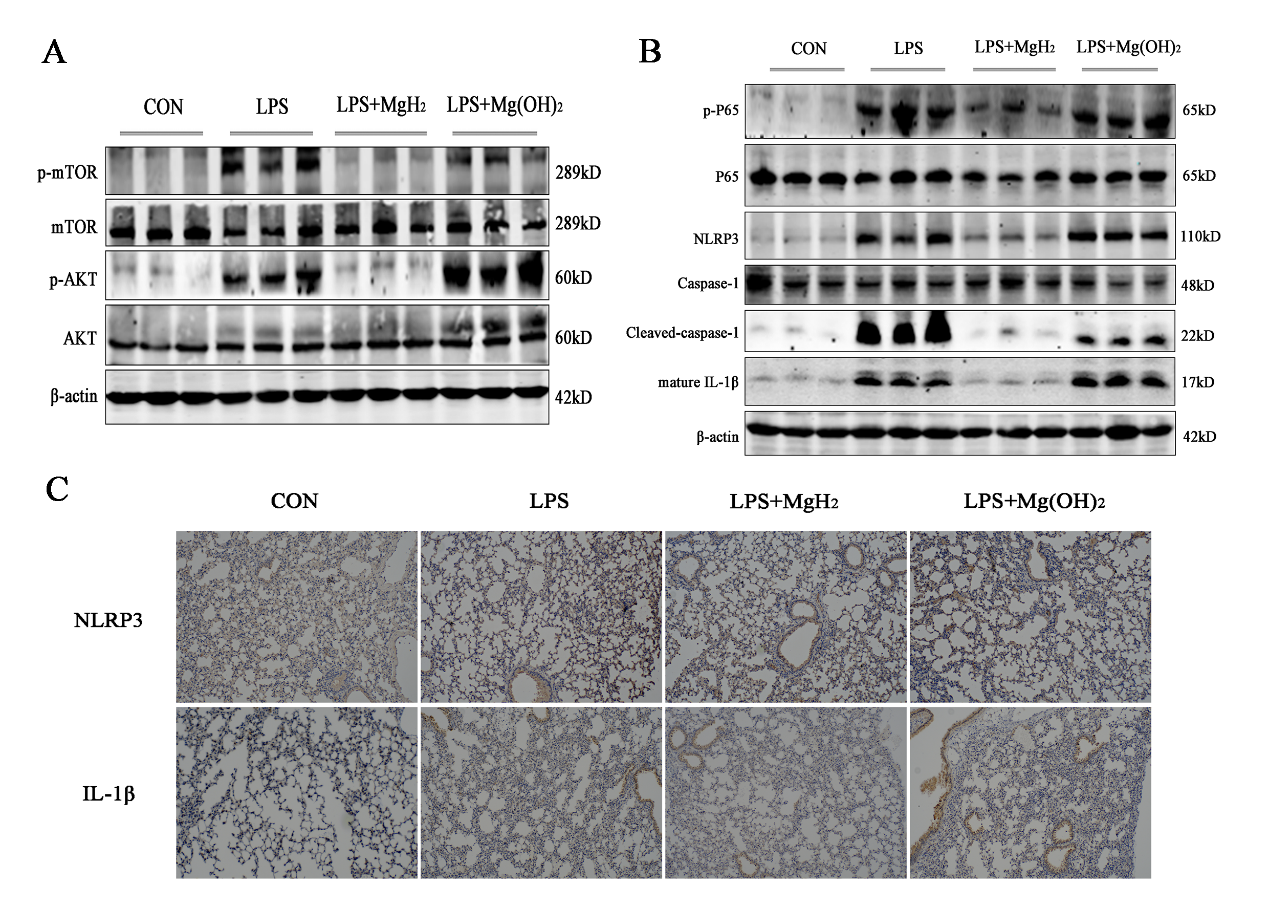


**Figure S3. MgH_2_ suppresses AKT/mTOR pathway and NF-κB/NLRP3/IL-1β pathway in endotoxemia mice. A-B.** MgH_2_ inhibits AKT/mTOR pathway and NF-κB/NLRP3/IL-1β pathway related proteins in vivo. **C.** NLRP3 and IL-1β immunohistochemistry staining in lung tissues (100X).

**Table S1. Primers used for reverse transcription-quantitative PCR.**

| Target gene | Forward primer | Reverse primer |
| --- | --- | --- |
| Mus β-actin | GGCTGTATTCCCCTCCATCG | CCAGTTGGTAACAATGCCATGT |
| Mus IL-1β | GCAACTGTTCCTGAACTCAACT | ATCTTTTGGGGTCCGTCAACT |
| Mus IL-6 | TAGTCCTTCCTACCCCAATTTCC | TTGGTCCTTAGCCACTCCTTC |
| Mus TNFα | AAGCCTGTAGCCCACGTCGTA | GGCACCACTAGTTGGTTGTCTTTG |
| Homo β-actin | CATGTACGTTGCTATCCAGGC | CTCCTTAATGTCACGCACGAT |
| Homo IL-1β | AGCTACGAATCTCCGACCAC | CGTTATCCCATGTGTCGAAGAA |
| Homo IL-6 | ACTCACCTCTTCAGAACGAATTG | CCATCTTTGGAAGGTTCAGGTTG |
| Homo TNFα | CCTCTCTCTAATCAGCCCTCTG | GAGGACCTGGGAGTAGATGAG |

IL, interleukin; TNF, tumor necrosis factor
